# Supplementary material for: Three-dimensional scene boundary representations for wall orientation and distance are represented distinctly in the human visual cortex
Source: PLoS Biol. 2026 Mar 25;24(3):e3003541. doi: 10.1371/journal.pbio.3003541 (PMC13043059; doi:10.1371/journal.pbio.3003541)
Supplement: S1 Fig — The description for the procedures of the calculation can be found in Methods of the main text. (DOCX) [file pbio.3003541.s001.docx]

**
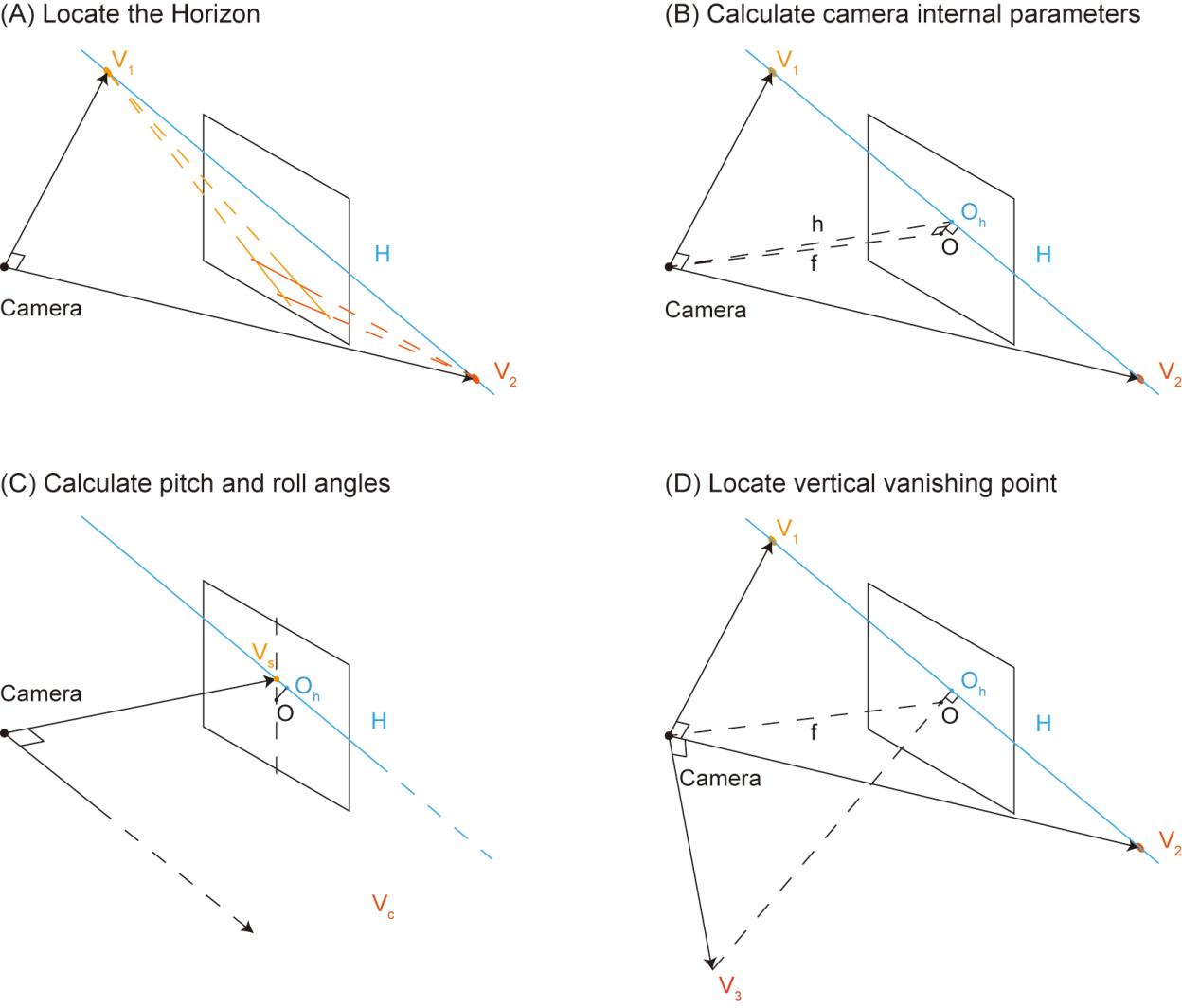
**

**Supplementary Figure 1**

The detailed illustration for calculating internal and external parameters. The description for the procedures of the calculation can be found in Methods of the main text.
