# Supplementary material for: Three-dimensional scene boundary representations for wall orientation and distance are represented distinctly in the human visual cortex
Source: PLoS Biol. 2026 Mar 25;24(3):e3003541. doi: 10.1371/journal.pbio.3003541 (PMC13043059; doi:10.1371/journal.pbio.3003541)
Supplement: S2 Fig — Each plot shows the RSA results after adding one of the alternative models (mean depth model, 3D surface model, and fwall model), with the alternative model’s partial correlation represented by the yellow box. A, Mean depth model. B, 3D surface model (surface orientation × distance) [16]. C, fwall model [15]. Asterisks denote significant results in the one-tailed t test against chance level. * q < 0.05; ** q < 0.01, *** q < 0.001. The data underlying this figure can be found at https://doi.org/10.17605/OSF.IO/UXWR4. (DOCX) [file pbio.3003541.s002.docx]

**
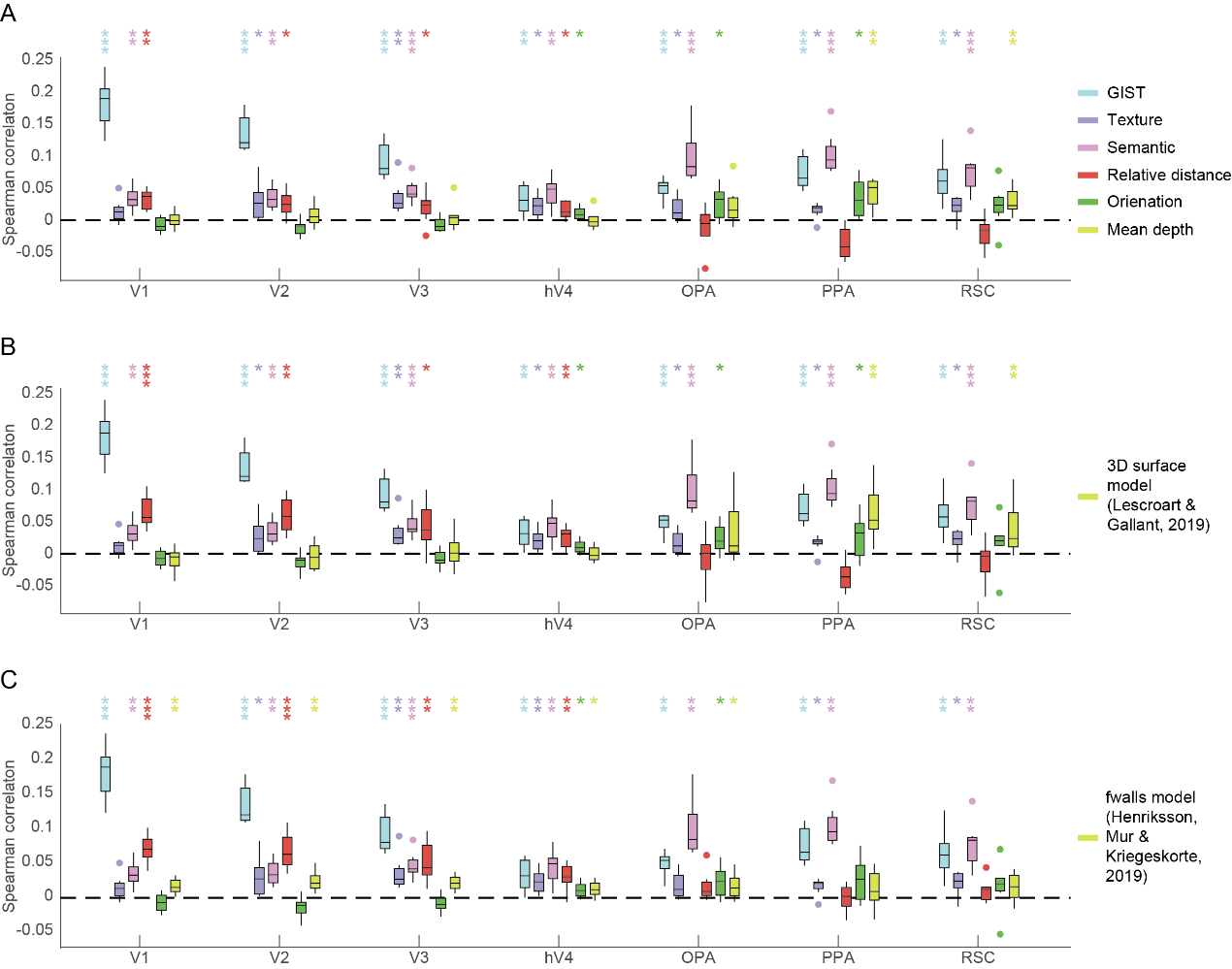
**

**Supplementary Figure 2**

The partial correlation analyses of alternative layout models for the NSD experiment. Each plot shows the RSA results after adding one of the alternative models (mean depth model, 3D surface model, and fwall model), with the alternative model’s partial correlation represented by the yellow box. **A,** Mean depth model. **B,** 3D surface model (surface orientation × distance) (Lescroart & Gallant, 2019). **C,** fwall model (Henriksson et al., 2019). Asterisks denote significant results in the one-tailed t-test against chance level. * *q* < 0.05; ** *q* < 0.01, *** *q* < 0.001. The data underlying this figure can be found at https://doi.org/10.17605/OSF.IO/UXWR4
