# Supplementary material for: Three-dimensional scene boundary representations for wall orientation and distance are represented distinctly in the human visual cortex
Source: PLoS Biol. 2026 Mar 25;24(3):e3003541. doi: 10.1371/journal.pbio.3003541 (PMC13043059; doi:10.1371/journal.pbio.3003541)
Supplement: S3 Fig — Searchlight-based partial correlation was performed and the results are displayed on the flattened cortical surfaces. (DOCX) [file pbio.3003541.s003.docx]

**
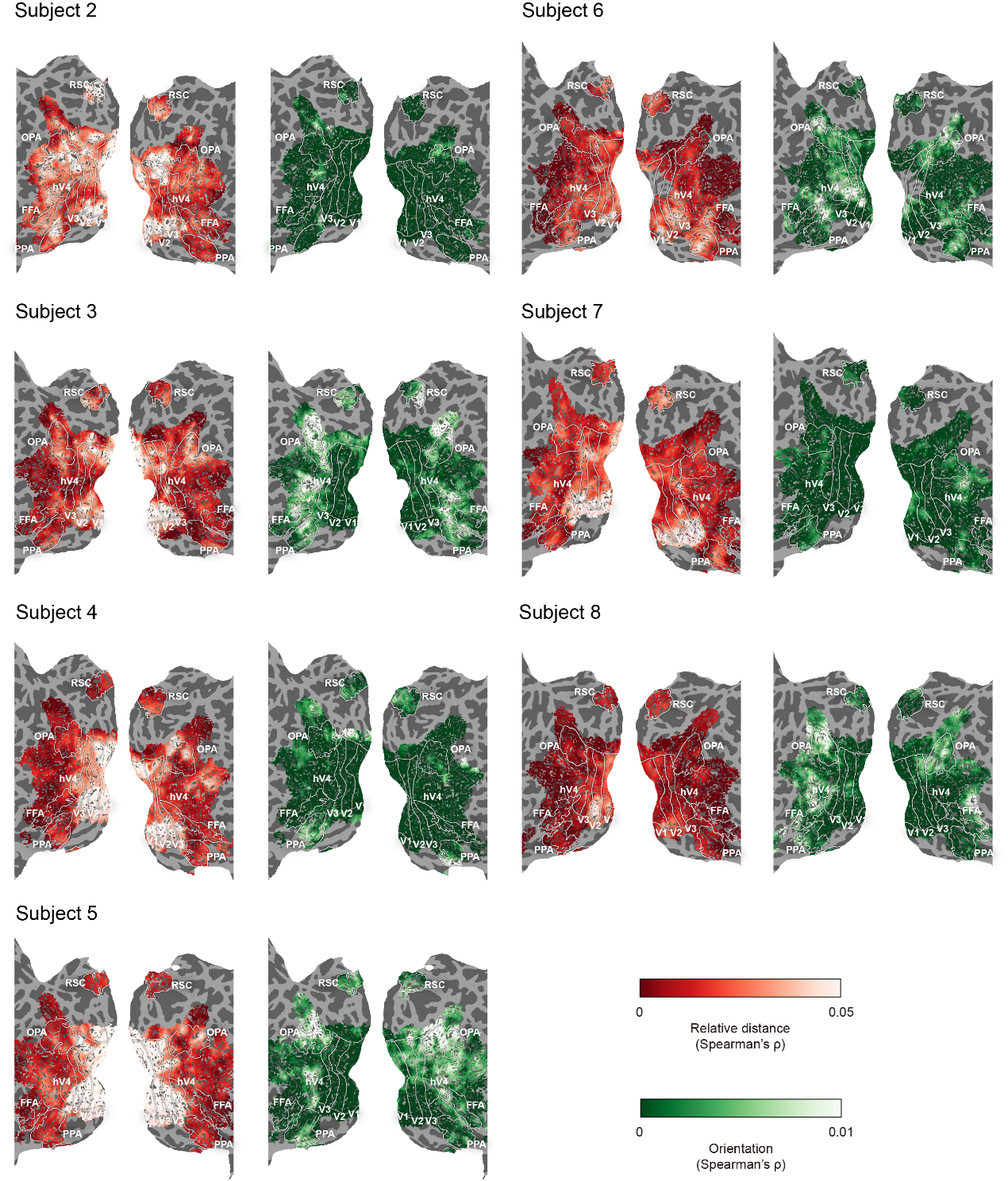
**

**Supplementary Figure 3**

Representations of layout in other seven NSD participants. Searchlight-based partial correlation was performed and the results are displayed on the flattened cortical surfaces.
