# Supplementary material for: Three-dimensional scene boundary representations for wall orientation and distance are represented distinctly in the human visual cortex
Source: PLoS Biol. 2026 Mar 25;24(3):e3003541. doi: 10.1371/journal.pbio.3003541 (PMC13043059; doi:10.1371/journal.pbio.3003541)
Supplement: S5 Fig — A and C, RSA results including the central contrast model in layout and texture discrimination tasks, respectively. B and D, RSA results including the object clutter model in layout and texture discrimination tasks, respectively. Asterisks denote significant results in the one-tailed t test against chance level. * q < 0.05; ** q < 0.01, *** q < 0.001. The data underlying this figure can be found at https://doi.org/10.17605/OSF.IO/UXWR4. (DOCX) [file pbio.3003541.s005.docx]

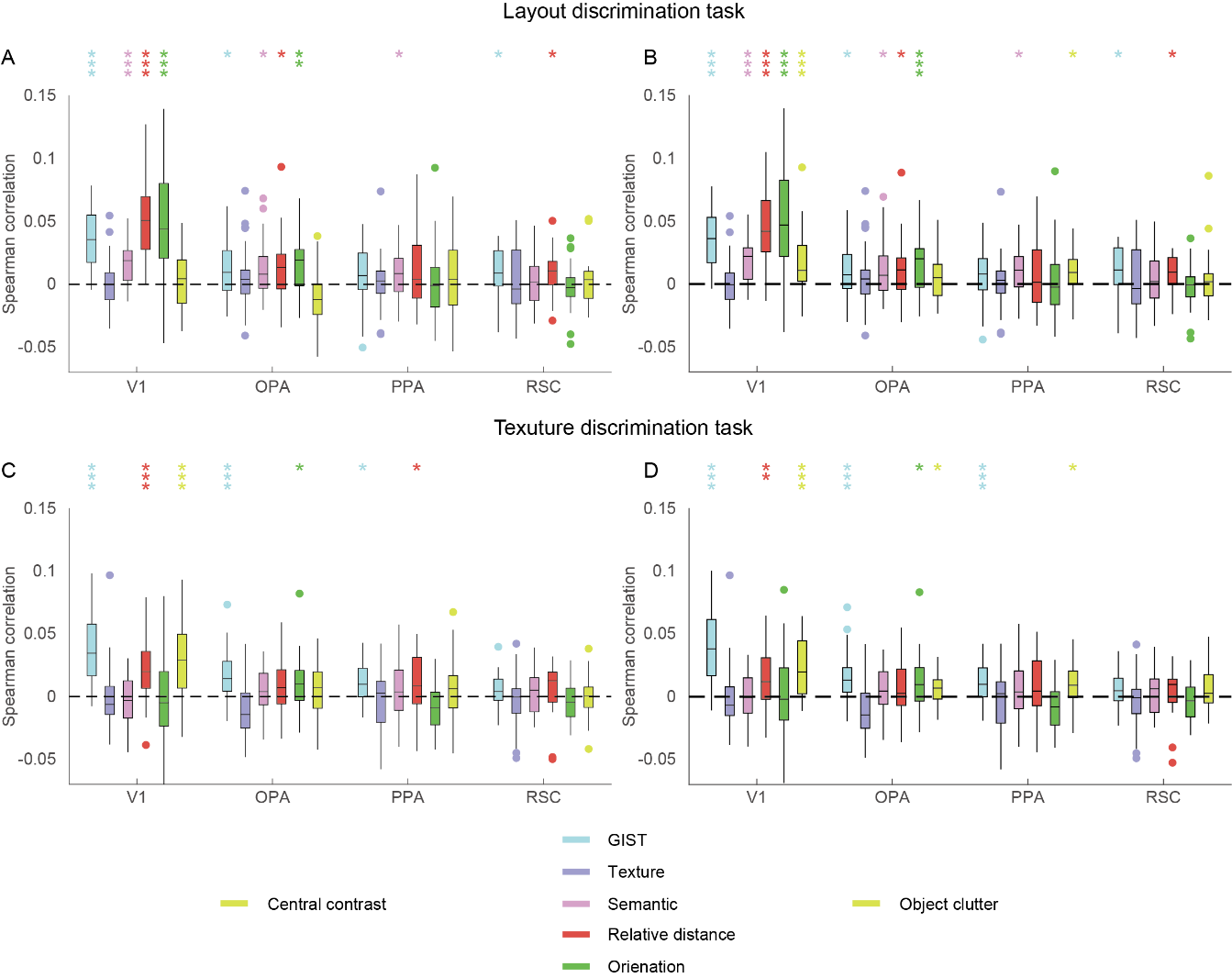


**Supplementary Figure 5**

The partial correlation analyses of alternative 2D feature models in the Matterport3D fMRI experiment. **A & C**, RSA results including the central contrast model in layout and texture discrimination tasks, respectively. **B & D**, RSA results including the object clutter model in layout and texture discrimination tasks, respectively. Asterisks denote significant results in the one-tailed t-test against chance level. * *q* < 0.05; ** *q* < 0.01, *** *q* < 0.001. The data underlying this figure can be found at https://doi.org/10.17605/OSF.IO/UXWR4
