# Supplementary material for: Three-dimensional scene boundary representations for wall orientation and distance are represented distinctly in the human visual cortex
Source: PLoS Biol. 2026 Mar 25;24(3):e3003541. doi: 10.1371/journal.pbio.3003541 (PMC13043059; doi:10.1371/journal.pbio.3003541)
Supplement: S6 Fig — The 2D visual, texture and semantic models were also involved in this partial correlation analysis but are not displayed for clarity. Statistical testing procedures are consistent with those in Fig 7 of the main text. The data underlying this figure can be found at https://doi.org/10.17605/OSF.IO/UXWR4. (DOCX) [file pbio.3003541.s006.docx]

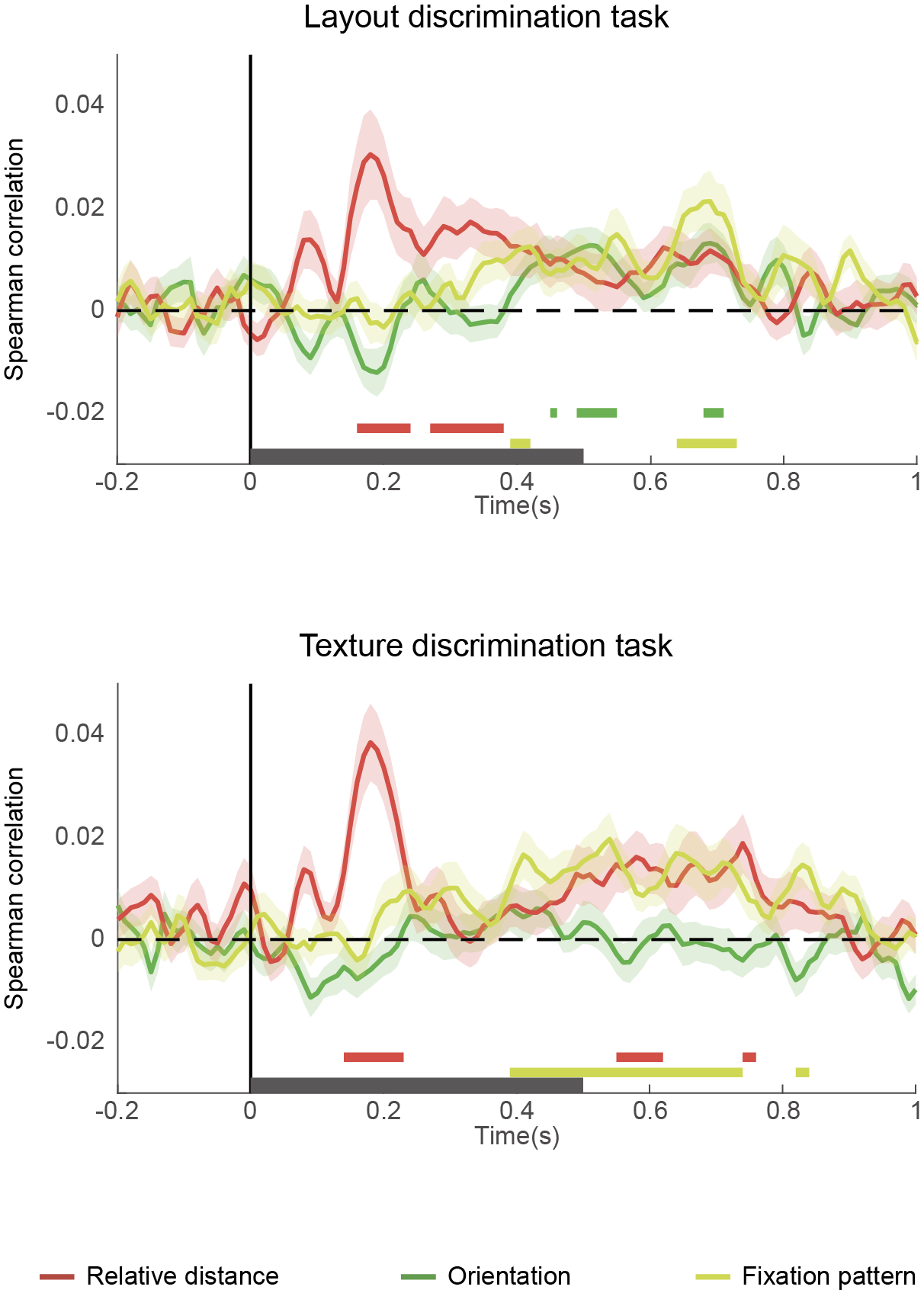


**Supplementary Figure 6**

The partial correlation analysis of alternative fixation model in the Matterport3D MEG experiment, incorporating the within-task farthest fixation patterns. The 2D visual, texture and semantic models were also involved in this partial correlation analysis but are not displayed for clarity. Statistical testing procedures are consistent with those in Figure 7 of the main text. The data underlying this figure can be found at https://doi.org/10.17605/OSF.IO/UXWR4
