# Supplementary material for: Three-dimensional scene boundary representations for wall orientation and distance are represented distinctly in the human visual cortex
Source: PLoS Biol. 2026 Mar 25;24(3):e3003541. doi: 10.1371/journal.pbio.3003541 (PMC13043059; doi:10.1371/journal.pbio.3003541)
Supplement: S7 Fig — The left plots show full correlations between 3D layout models and ROIs RDMs in Matterport3D fMRI experiment. Statistical testing procedures of left plots are consistent with those in Fig 6 of the main text. The right plots show full correlations between 3D layout models and occipital channel RDMs in Matterport3D MEG experiment. Statistical testing procedures of right plots are consistent with those in Fig 7 of the main text. A, Layout discrimination task. B, Texture discrimination task. C, Task-dependent enhancement of representation. The data underlying this figure can be found at https://doi.org/10.17605/OSF.IO/UXWR4. (DOCX) [file pbio.3003541.s007.docx]

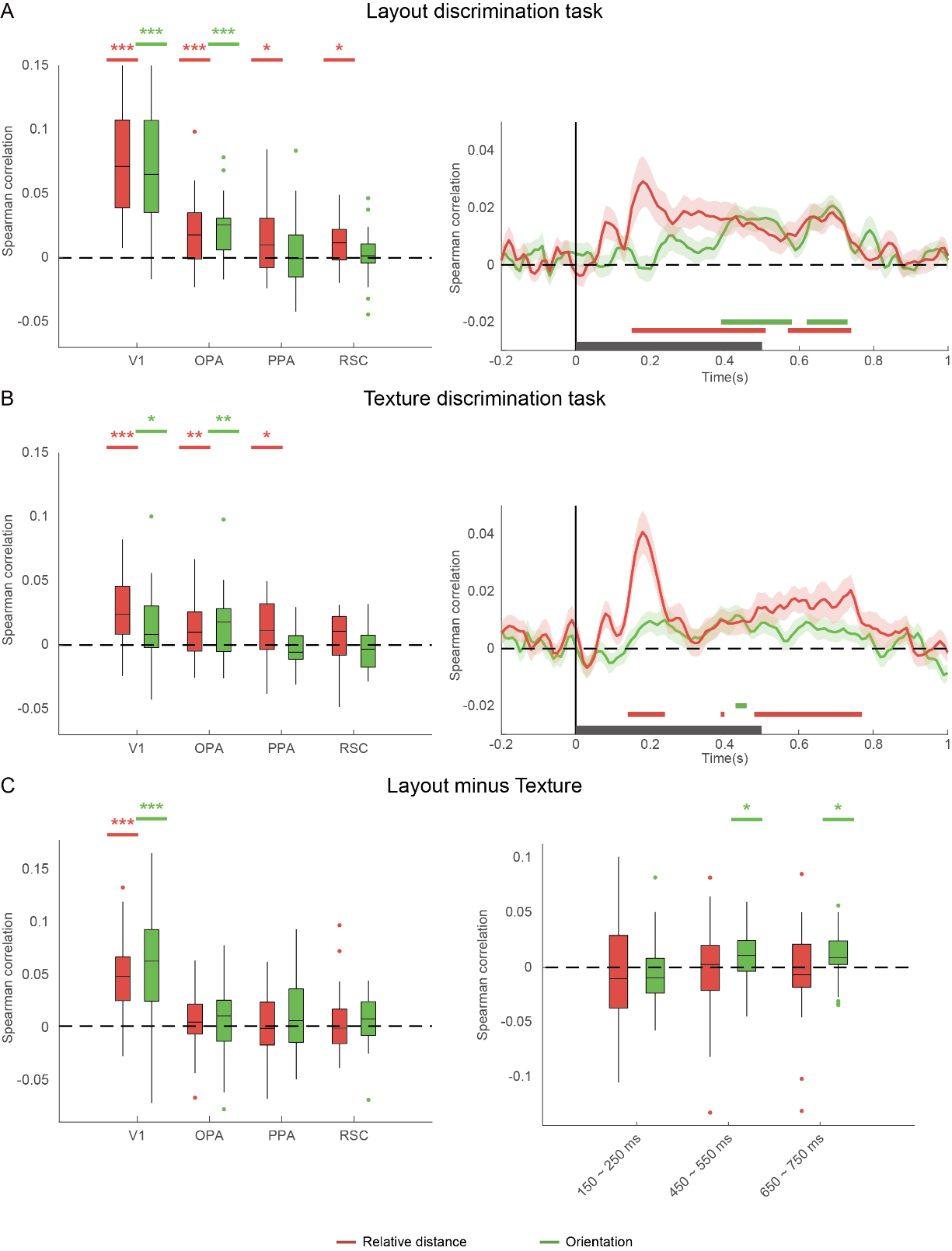


**Supplementary Figure 7**

The full Spearman correlation analysis of 3D layout models in Matterport3D fMRI and MEG experiments. The left plots show full correlations between 3D layout models and ROIs RDMs in Matterport3D fMRI experiment. Statistical testing procedures of left plots are consistent with those in Fig 6 of the main text. The right plots show full correlations between 3D layout models and occipital channel RDMs in Matterport3D MEG experiment. Statistical testing procedures of right plots are consistent with those in Fig 7 of the main text. **A**, Layout discrimination task. **B**, Texture discrimination task. **C**, Task-dependent enhancement of representation. The data underlying this figure can be found at https://doi.org/10.17605/OSF.IO/UXWR4
