# Supplementary material for: Three-dimensional scene boundary representations for wall orientation and distance are represented distinctly in the human visual cortex
Source: PLoS Biol. 2026 Mar 25;24(3):e3003541. doi: 10.1371/journal.pbio.3003541 (PMC13043059; doi:10.1371/journal.pbio.3003541)
Supplement: S1 Table — (DOCX) [file pbio.3003541.s008.docx]

**Supplementary Table 1**

*Partial correlations between 2D model RDMs and neural RDMs in layout discrimination task: Matterport3D fMRI experiment.*

| ROIs | Models | Mean partial corr. | Standard Dev. | *p* value  (FDR corrected) |
| --- | --- | --- | --- | --- |
| V1 | GIST | 0.035 | 0.023 | 0.000 |
|  | Texture | 0.000 | 0.021 | 0.608 |
|  | Semantic | 0.017 | 0.017 | 0.000 |
|  | Relative distance | 0.052 | 0.034 | 0.000 |
|  | Orientation | 0.052 | 0.051 | 0.000 |
| OPA | GIST | 0.010 | 0.020 | 0.021 |
|  | Texture | 0.007 | 0.027 | 0.130 |
|  | Semantic | 0.012 | 0.023 | 0.014 |
|  | Relative distance | 0.013 | 0.024 | 0.011 |
|  | Orientation | 0.016 | 0.023 | 0.001 |
| PPA | GIST | 0.007 | 0.021 | 0.083 |
|  | Texture | 0.001 | 0.022 | 0.492 |
|  | Semantic | 0.008 | 0.018 | 0.034 |
|  | Relative distance | 0.011 | 0.030 | 0.054 |
|  | Orientation | -0.001 | 0.030 | 0.675 |
| RSC | GIST | 0.011 | 0.018 | 0.014 |
|  | Texture | 0.002 | 0.027 | 0.492 |
|  | Semantic | 0.004 | 0.021 | 0.301 |
|  | Relative distance | 0.009 | 0.019 | 0.035 |
|  | Orientation | -0.002 | 0.020 | 0.778 |
