# Supplementary material for: Three-dimensional scene boundary representations for wall orientation and distance are represented distinctly in the human visual cortex
Source: PLoS Biol. 2026 Mar 25;24(3):e3003541. doi: 10.1371/journal.pbio.3003541 (PMC13043059; doi:10.1371/journal.pbio.3003541)
Supplement: S3 Table — (DOCX) [file pbio.3003541.s010.docx]

**Supplementary Table 3**

*Task-dependent enhancement of representation, calculated as the difference between partial correlation coefficients for the two tasks (layout minus texture): Matterport3D fMRI experiment.*

| ROIs | Models | Mean partial corr. | Standard Dev. | *p* value  (FDR corrected) |
| --- | --- | --- | --- | --- |
| V1 | GIST | -0.005 | 0.032 | 0.634 |
|  | Texture | 0.003 | 0.035 | 0.756 |
|  | Semantic | 0.018 | 0.027 | 0.007 |
|  | Relative distance | 0.029 | 0.039 | 0.004 |
|  | Orientation | 0.053 | 0.055 | 0.000 |
| OPA | GIST | -0.007 | 0.033 | 0.626 |
|  | Texture | 0.017 | 0.030 | 0.020 |
|  | Semantic | 0.006 | 0.026 | 0.626 |
|  | Relative distance | 0.005 | 0.028 | 0.634 |
|  | Orientation | 0.005 | 0.033 | 0.634 |
| PPA | GIST | -0.006 | 0.025 | 0.626 |
|  | Texture | 0.006 | 0.033 | 0.626 |
|  | Semantic | 0.003 | 0.029 | 0.708 |
|  | Relative distance | -0.001 | 0.035 | 0.878 |
|  | Orientation | 0.007 | 0.037 | 0.626 |
| RSC | GIST | 0.006 | 0.022 | 0.626 |
|  | Texture | 0.005 | 0.036 | 0.708 |
|  | Semantic | -0.001 | 0.028 | 0.878 |
|  | Relative distance | 0.004 | 0.033 | 0.708 |
|  | Orientation | 0.003 | 0.025 | 0.708 |
