# Supplementary material for: Three-dimensional scene boundary representations for wall orientation and distance are represented distinctly in the human visual cortex
Source: PLoS Biol. 2026 Mar 25;24(3):e3003541. doi: 10.1371/journal.pbio.3003541 (PMC13043059; doi:10.1371/journal.pbio.3003541)
Supplement: S1 Text — (DOCX) [file pbio.3003541.s011.docx]

Three-dimensional scene boundary representations for wall orientation and distance are represented distinctly in the human visual cortex

Yichen Wu and Sheng Li

# Supplementary Methods

## NSD experiment

### Alternative layout models

#### *Mean depth model*

The mean depth model was calculated in the same way as in the layout relative distance model (i.e., counting the proportion of side wall pixels). Effectively, the mean depth model can be considered as a special case of relative distance model that the entire image serves as a single bin.

#### *3D surface model (surface orientation and distance)*

Inspired by the full 3D structural model in Lescroart & Gallant (2019), this model measures surface orientation and distance in the scene image at pixel level. Based to the reconstruction of boundaries, 3D surface model focuses only on the surface orientation and distance of walls, including ceiling, floor, and side walls. We used the reconstructed aerial view annotations to compute the orientation for each pixel in the reconstructed layout segmentation map. The surface distance is estimated using the proportion of side wall pixels in each column as the layout relative distance model does.

3D surface model uses 50 feature channels (5 orientation bins × 10 distance bins). The boundaries of the distance bins are logarithmically spaced from 0% to 100% of side wall pixels. The orientation bins include 3 horizontal bins and 2 vertical bins. The horizontal bins have the central normal vector that faces right (yaw: 0°), backward (yaw: 90°), and left (yaw: 180°). The vertical bins have the central normal vector that faces down (pitch: 0°) and up (pitch: 180°). The yaw angle of 0° and pitch angle of 0° align with the observer's right and upward directions, respectively. Angles increase with clockwise rotation. Pixels belonging to the ceiling and floor in the layout segmentation map are assigned to the bins of facing up and down, respectively. Side wall pixels are partitioned according to the following equation:

$$F_{p,i}=max\left( 0,\frac{\sigma-abs(\theta_{p}-\theta_{i}^{c})}{\sigma} \right)$$

where σ is the angular width of each horizontal bin, $\theta_{i}^{c}$ is the center yaw angle of the *i*-th bin, and $\theta_{p}$ represents the yaw angle of normal vector at a given pixel *p*. In this model, σ is set as 90°. In the calculation of feature channel responses, scene pixels are initially masked and partitioned according to their distance bins. Then, the contribution of each pixel to the orientation bins $F_{p,i}$ are computed using the above equation. The final response for each feature channel is derived by summing the contributions of all partitioned pixels in the distance bin.

#### *fwall model*

Inspired by the work of Henriksson et al. (2019), this model uses five feature channels to represent the presence or absence of the ceiling, floor, left wall, right wall, and back wall. We defined the side walls using the yaw angle of their normal vectors: a yaw angle below 45° as the left wall, 45° to 135° as the back wall, and over 135° as the right wall.

## Matterport3D fMRI experiment

### Alternative 2D feature models

#### *Central contrast model*

The central contrast model is computed as the standard deviation of pixel intensities within a central circular region (2° radius) for each image in gray scale.

#### *Object clutter model*

We leveraged the instance labels provided in the Matterport3D dataset to quantify the number of objects within each of the five bins used in the relative distance model. Only object instances with a pixel area exceeding 0.1% of the total image area were included.

### Alternative layout models

#### *Layout precise distance model*

The precise distance of the walls in the Matterport3D image set can be computed using the depth maps provided by the dataset and the wall segmentation maps from the Matterport3D-layout dataset (Zhang et al., 2020). Therefore, in the Matterport3D fMRI experiment, we were able to generate precise distances between the observer and the walls. In the model, we divided the entire field of view into 5 bins evenly. We adopted the same equation for calculating feature channel response as in the layout orientation model:

$$F_{i}\left( \varphi\right)=max\left( 0,\frac{\sigma-abs(\varphi-\varphi_{i}^{c})}{\sigma} \right)$$

$$R_{i}=\int D(\varphi)F_{i}(\varphi)d\varphi$$

where *σ* is the angular width of each bin, $\varphi_{i}^{c}$is the center of the *i*-th bin, and *φ* represents any direction in the field of view. The responses of the bins are computed by integrating over the entire field of view, where $F_{i}(\varphi)$ is the soft-histogram function for the *i*-th bin and $D(\varphi)$ corresponds to the precise distance of the wall at the direction *φ* in meters.

#### *Mean depth model*

The mean depth model is calculated in meters as a precise distance model with the entire field of view serving as a single bin. This method emphasizes the importance of the wall distance at the center of the field of view within the scene image.

The RDMs of all alternative 2D feature and 3D layout models in Matterport3D fMRI experiment were constructed by calculating the Cityblock distance.

## Matterport3D MEG experiment

### Eye tracking setup

Eye movements were recorded using an EyeLink 1000+ desktop-mount eye tracker with a sampling rate of 1000 Hz. Participants’ eye movements were calibrated and validated before the experiment. A velocity threshold of 35°/sec and an acceleration threshold of 9500°/sec^2^ were used for saccade detection.

### Alternative fixation pattern model

For each participant, we identified the gaze point with the maximum displacement from the central fixation cross as the farthest fixation position within each trial. To ensure a stable estimation of gaze behavior, for each participant and each task, we averaged the coordinates of these farthest fixation points across the three trials in which a specific image was presented, resulting in a single mean farthest fixation position per image. The eye-movement RDM was then constructed by calculating the Euclidean distance between these farthest fixation coordinates for each pair of images.

# Supplementary Results

## Matterport3D fMRI experiment

### Partial correlations between 2D image model RDMs and neural RDMs

As shown in Fig 6 of main text, in the layout discrimination task, we found significant partial correlations of the GIST model with neural activity in V1, OPA, and RSC (V1: t(29) = 8.48, *q* < 0.001; OPA: t(29) = 2.56, *q* = 0.021; RSC: t(22) = 2.89, *q* = 0.014; one-tailed, FDR corrected), as well as significant partial correlations of the semantic model with V1, PPA, and OPA (V1: t(29) = 5.37, *q* < 0.001; PPA: t(29) = 2.33, *q* = 0.034; OPA: t(29) = 2.85, *q* = 0.014; one-tailed, FDR corrected). In the texture discrimination task, only the GIST model showed significant partial correlations with neural activity in V1, PPA, and OPA (V1: t(29) = 7.44, *q* < 0.001; PPA: t(29) = 4.16, *q* < 0.001; OPA: t(29) = 4.41, *q* < 0.001; one-tailed, FDR corrected). Task-dependent enhancements were computed by subtracting the correlation coefficient of texture task from the correlation coefficient of layout task (Fig 6C). We observed enhanced semantic model correlations in V1 (t(29) = 3.66, *q* = 0.003, two-tailed, FDR corrected) and enhanced texture model correlations in OPA (t(29) = 3.12, *q* = 0.010, two-tailed, FDR corrected) when participants performed the layout discrimination task.
