# Supplementary material for: Three-dimensional scene boundary representations for wall orientation and distance are represented distinctly in the human visual cortex
Source: PLoS Biol. 2026 Mar 25;24(3):e3003541. doi: 10.1371/journal.pbio.3003541 (PMC13043059; doi:10.1371/journal.pbio.3003541)
Supplement: Attachment — Submitted filename: Response-letter-final.docx [file pbio.3003541.s014.docx]

**Response to the General Comments.**

1. **Concerns related to eye movement.**

We thank the reviewers for raising the important concern regarding potential confounds from eye movements. To directly address this issue, we conducted additional analyses of available eye-tracking data from the NSD dataset, reported eye-tracking results from our Matterport3D MEG experiment, and performed control RSA analyses incorporating eye-movement measures.

First, in the NSD experiment (Allen et al., 2022), participants were explicitly instructed to maintain central fixation. According to the description of the dataset, fixation stability was emphasized through pre-scanning training and reminders at the beginning of each scanning session. To verify the fixation compliance, we analyzed the available eye-tracking data from six participants across several sessions. As shown in Figure R1, we quantified both the number of fixations and the maximum displacement from the central fixation point within each trial (3-s stimulus duration). Participants exhibited highly stable fixation behavior: the mean maximum displacement remained within 2° for all subjects (Figure R2A). Given that the images subtended 8.4°, these results indicate that gaze remained largely confined to the central region of the stimuli. The present study focused on a subset of approximately 2,000 indoor images. Because only a limited number of these trials contained eye-tracking recordings, the data were insufficient to construct a reliable eye-movement RDM for RSA analyses. Nevertheless, the overall fixation stability observed across the available eye movement data provides strong evidence that participants adhered closely to fixation instructions.

Second, in the Matterport3D experiments, stimulus presentation duration was limited to 500 ms to minimize opportunities for exploratory eye movements. Participants fixated a central cross before each trial and were instructed to maintain fixation during stimulus presentation. Eye movements were not recorded in the fMRI experiment due to technical complexity and procedural constraints associated with the task-load of the participants (i.e., additional anatomical and localizer scans as compared to the MEG experiment). However, eye-tracking data were collected from all participants in the MEG experiment. Although originally used only to monitor fixation quality, we now perform further analyses on these data. As shown in Figure R2B, the mean farthest fixation distance from the central fixation during a trial was 1.330° (standard deviation: 0.527°). Relative to the stimulus size (9.5° × 7.6°), this indicates strong compliance with fixation instructions and minimal large-scale gaze shifts.

Third, we observed systematic patterns in small-scale eye movements during the MEG experiment. Specifically, fixation patterns were consistent within- and cross-participants across repeated presentations of the same image within the same task (Figure R2C), as well as the within-participants similarity across tasks (Figure R2D). Such consistency may reflect shared covert attention patterns, consistent with prior work showing that microsaccade direction can index the location of attentional focus (Engbert & Kliegl, 2003; Hafed & Clark, 2002; Yuval-Greenberg et al., 2014). To rigorously test whether these patterns contributed to the neural RSA results, we constructed an eye-movement RDM based on the farthest fixation location. The RDM was constructed by calculating the Euclidean distance between the farthest fixation positions for each image pair and incorporated as a control variable in the MEG partial RSA analyses. Results showed that although farthest fixation patterns could be decoded late in the trial (after stimulus offset), the key variables of interest—boundary relative distance and orientation—remained significant after partialling out the eye-movement RDM (Figure R2E). These findings suggest that the observed neural representations of scene layout cannot be explained by gaze-related variance in neural activity.

In summary, behavioral and eye-tracking metrics demonstrate stable central fixation across datasets. Control RSA analyses further confirm that the spatiotemporal dynamics of relative distance and orientation representations are robust to residual eye movement patterns. Considering (i) the direct eye-movement control analyses in the MEG experiment, (ii) the strong convergence between fMRI and MEG representational structures, and (iii) the highly similar paradigms and fixation instructions between the two experiments, we suggest that the results in the Matterport3D fMRI experiment reflect stable cortical representations of scene geometry rather than transient gaze shifts. In the revised manuscript, we have incorporated descriptions of the MEG eye-tracking procedure and the corresponding control RSA analyses in the appropriate sections of the Results (p. 14), Supplementary Methods, and Supplementary Figure 7.

**Figure R1**

Statistical characterization of eye movements in the NSD dataset. **Left**, Probability density functions (PDFs) of the number of fixations per trial for each participant. **Middle**, PDFs of the distance between the central fixation point and the farthest fixation location within each trial. **Right**, Scatter plots showing the spatial distribution of farthest fixation locations across trials. Stimuli subtended 714 × 714 pixels on the display.

**Figure R2**

Fixation stability and representational similarity of fixation patterns. **A**, Mean number of fixations and mean farthest fixation distance per trial in the NSD dataset. Each dot represents one participant. **B**, Mean number of fixations and mean farthest fixation distance per trial in the Matterport3D MEG experiment. Each dot represents one participant. **C**, Within-task similarity of farthest fixation patterns in the Matterport3D MEG experiment. Left: Within-subject similarity. Each image was presented three times within a task; RSA was computed between the farthest fixation RDM from one presentation and the average RDM from the remaining two presentations, followed by group averaging. Right: Between-subject similarity, computed by correlating each participant’s average farthest fixation RDM with the group-average RDM derived from the remaining participants. **D**, Cross-task similarity of farthest fixation patterns for each participant of the Matterport3D MEG experiment, obtained by correlating the averaged fixation RDMs from the layout discrimination and texture discrimination tasks. **E**, Partial RSA results incorporating within-task farthest fixation patterns as a control variable in the Matterport3D MEG experiment. Statistical procedures follow those reported in Figure 7 of the main text.

2. **Engagement of early visual cortex (V1) in orientation representation.**

We thank the reviewers for raising this important point. We agree that clarifying the contribution of early visual cortex (including V1) to boundary orientation representation is essential, and we have substantially expanded the Discussion section (p. 18-19) in the revised manuscript accordingly.

Boundary orientation is essential for reconstructing the 3D structure of environmental layouts. Recovering 3D structure from natural images requires the integration of multiple depth cues—including linear perspective, shading, texture gradients, and binocular disparity—across levels of the visual hierarchy, rather than simple extraction of local 2D edge features.

To clarify the mechanisms underlying layout processing, we relate our findings to established frameworks in depth perception. Under task-irrelevant conditions, we propose that early visual cortex contributes by representing local spatial structures and image-based depth cues that serve as inputs for estimating boundary orientation. This interpretation aligns with prior evidence showing that early visual cortex encodes monocular depth cues such as linear perspective, shading, and texture (Dövencioğlu et al., 2013; Georgieva et al., 2008; Gerardin et al., 2010; Welchman et al., 2005). In classical depth-processing models, early visual cortex participates in cue-specific encoding, whereas the integration of multiple cues into a coherent 3D percept occurs in higher-level region V3B (Welchman, 2016). Given the anatomical and functional proximity between OPA and V3B (Epstein & Baker, 2019), our findings suggest that OPA may similarly integrate multiple depth-related signals—including area-based distance information processed in early visual cortex—into more abstract structural representations of 3D layout.

Importantly, the task-dependent enhancement observed in early visual cortex may arise from at least two, potentially complementary mechanisms. First, it may reflect feedback modulation from higher-level scene-processing areas when layout information becomes behaviorally relevant. Previous work has shown that depth structure can reshape activity patterns in early visual cortex and that such modulation is strongly influenced by attention and contextual feedback (Fang et al., 2008; He et al., 2015; Murray et al., 2006). In this context, early visual cortex may serve as a high-resolution representational “sketchpad” that supports fine-grained spatial encoding when layout becomes behaviorally relevant. When navigation-related judgments are required, top-down feedback may re-engage early visual cortex to enhance the precision of boundary-related features.

Second, the enhancement may also reflect the short-term maintenance of depth cue-related features within early visual cortex. A growing body of evidence suggests that early visual areas can sustain sensory-like representations during visual working memory and imagery (Bainbridge et al., 2021; Dijkstra et al., 2018, 2019; Harrison & Tong, 2009; Jia et al., 2021; Kwak & Curtis, 2022; Pasternak & Greenlee, 2005; Rademaker et al., 2019; Serences et al., 2009; Xie et al., 2020). Navigation tasks require transient retention of boundary orientation and spatial structure, and early visual cortex may serve as a high-resolution representational buffer that maintains task-relevant depth cues over short intervals. Both feedback-driven gain modulation and local maintenance mechanisms would lead to enhanced encoding in early visual cortex and its detectability under task-relevant conditions.

At the same time, we do not exclude alternative or complementary explanations. Task-driven attention to specific locations or orientations in the 2D images, as well as memory-related maintenance of spatially localized information, may also contribute to late activity patterns in early visual cortex. Although we controlled for local orientation and spatial frequency information using the GIST model, residual spatially localized features may still covary with 3D layout properties. We have noted this possibility in the revised Discussion and suggest that future studies employing orientation-filtered natural images or more selective feature manipulations may help further disentangle these contributions.

3. **Mathematical concerns regarding partial correlation in RSA analyses.**

We thank the reviewers for their thoughtful comments on the use of partial correlation in our Representational Similarity Analysis (RSA). We appreciate the concern regarding the negative correlations observed in some partial RSA results and have taken several steps to clarify both the statistical rationale and the interpretation of these findings. In the revised manuscript, we now include the full (Spearman) RSA results—without partialing out other models—in the Supplementary Figures 8 and 9 and provide additional methodological clarification (p. 6, 27, 32, and 34) as outlined below.

First, partial correlation is a well-established analytical approach in RSA that allows researchers to estimate the unique variance explained by a target model while statistically controlling for shared variance with other models. This approach has been widely adopted in studies using complex or naturalistic stimuli, where multiple feature dimensions are inherently correlated (e.g., Cichy et al., 2019; Giari et al., 2020; Kaiser et al., 2022; Lu & Golomb, 2025; Reddy et al., 2021; Xu et al., 2024). For example, Kaiser et al. (2022) modeled fMRI neural dissimilarity using a semantic RDM while controlling for emotional, visual, and auditory models via partial Spearman correlation. Similarly, Lu and Golomb (2025) used partial correlation RSA to dissociate EEG representations of real-world size, depth, and retinal size. These precedents illustrate the appropriateness of partial correlation for disentangling overlapping representational dimensions in multivariate neural analyses.

Second, we clarify that the negative correlations observed in the MEG partial RSA are not methodological artifacts but reflect a known statistical phenomenon in multivariate modeling, often described as a suppressor effect. When predictors (e.g., boundary relative distance and orientation) share substantial variance, partial correlation estimates the relationship between the residual components of each predictor after removing shared variance. If one predictor accounts for a large proportion of the shared variance with neural responses, the remaining residual variance of another predictor may exhibit a reduced or even sign-reversed relationship with the data. Importantly, this does not imply that the feature is negatively represented in the neural signal; instead, it arises from the removal of shared variance during partial correlation, which isolates the feature’s unique explanatory component. Similar sign reversals between full and partial RSA have been reported in prior work using comparable analytic frameworks (e.g., Lu & Golomb, 2025; Figure R3).

To ensure full transparency and facilitate interpretation, we now provide the full (Spearman) RSA results without partialing out other models (Figure R4; Supplementary Figures 8 & 9). These analyses demonstrate that both boundary relative distance and orientation exhibit non-negative correlations with neural responses when examined individually. The partial RSA results presented in the main text therefore serve a complementary purpose: they isolate the unique contribution of each model while accounting for shared variance across feature dimensions. Notably, the results suggest that relative distance accounts for a larger portion of the shared representational structure. After controlling for this shared component, the residual contribution of orientation is substantially reduced, indicating that part of its early-stage positive correlation with neural activity reflects overlapping variance with distance.

**Figure R3**

Illustration of sign reversals between full and partial RSA analyses (adapted from Lu & Golomb, 2025). This EEG study investigated representations of real-world size, depth, and retinal size during object viewing. The upper panel shows full RSA results based on direct correlations between model and neural RDMs. The lower panel shows partial correlation RSA results after controlling for shared variance across models. A sign reversal in the real-world size representation around ~100 ms illustrates how suppressor effects can emerge when highly correlated predictors are statistically dissociated. This example is provided to clarify the statistical interpretation of negative partial correlations observed in the present study.

**Figure R4**

Full RSA results for 3D layout models in the Matterport3D fMRI and MEG experiments. **Left**: Full Spearman correlations between 3D layout model RDMs and neural RDMs across regions of interest in the fMRI experiment. **Right**: Full Spearman correlations between 3D layout model RDMs and MEG RDMs derived from occipital sensors. These analyses do not partial out other feature models and are provided to complement the partial correlation RSA results reported in the main text (Figures 6 and 7). Statistical testing procedures and significance conventions are identical to those used in the main text.

**Point by Point Response to Reviewers’ Comments.**

**Reviewer 1**

*Major*

1) *The authors control for 2D image features using various models. However, these models (e.g., GIST) are far from perfect and it is likely that there are residual visual differences that were not accounted for. Indeed, the variables of interest (e.g., wall distance and orientation) are themselves defined visually (e.g., orientation, known to be encoded in V1), such that fully accounting for visual feature differences would presumably eliminate any effect of interest? By controlling for image features, would you argue that the results do not reflect the processing of image features then? Finally, if the goal is to regress out image features, did you consider creating RDMs using feature representations in the various layers of DNNs (e.g., AlexNet), which should better capture visual features than GIST etc.?*

We thank the reviewer for raising this important issue. We agree that spatial layout properties, such as boundary distance and orientation, are inherently grounded in visual input, and we do not claim that the neural representations identified here are independent of visual feature processing. On the contrary, we assume that the visual system relies on features such as figure-ground structure, local orientation, and texture to infer spatial layout. Our goal in including 2D image models was therefore not to eliminate visual contributions, but to test whether layout-related representations explain unique variance beyond what can be accounted for by standard low- and mid-level visual feature models (e.g., GIST and texture).

Importantly, controlling for image-based models does not imply that the observed neural effects are unrelated to visual processing. Rather, it addresses a more specific question: whether the representational structure associated with boundary distance and orientation can be reduced to simple image-level similarity captured by established visual models. The persistence of significant layout effects after controlling for GIST, texture, and semantic models suggests that the neural representations reflect structured spatial layout information that cannot be fully explained by conventional image statistics alone. This analytical logic follows established practices in studies of navigational affordance and scene layout representation, where visual models are included to test and reject the null hypothesis that navigation-related effects arise solely from low-level visual similarity (e.g., Bonner & Epstein, 2017; Henriksson et al., 2019; Lescroart & Gallant, 2019). We have further clarified this rationale in the Results section (p. 6) of the revised manuscript.

Regarding the reviewer’s suggestion to use Deep Neural Network (DNN) features as additional controls, we agree that DNN-based representations can provide powerful summaries of visual structure. However, we intentionally prioritized interpretable low- and mid-level feature models (e.g., GIST and texture) for the following reasons. Low-level models such as GIST have well-characterized feature spaces, allowing clearer interpretation of what variance is being controlled. In contrast, it is difficult to precisely isolate what information is represented in each layer of a DNN. Representations in intermediate and higher layers of DNNs are known to capture spatial layout and navigationally relevant properties similar to those investigated in the present study. For example, Bonner and Epstein (2018) demonstrated that navigation-related layout information emerges in layers 2 through 6 of a deep CNN. Including such layers as control models could therefore partial out the neural variance associated with our variables of interest (boundary distance and orientation), increasing the risk of over-controlling and producing false negatives. By using established image-statistic models, we aimed to control for conventional visual similarities while preserving the ability to detect higher-level layout representations.

2) *There is a close link between eye movements and fMRI responses to natural scenes (e.g., O'Connell & Chun, Nat Comm 2018). It was not clear if/how eye movements were controlled in the present work. It is quite possible that participants fixated different parts of a scene depending on its layout (e.g., near vs far). This is particularly concerning for the NSD study, where scenes were presented for 3 seconds. The NSD does include some eye tracking sessions - perhaps these can be analyzed to exclude this. Alternatively, you could run a separate eye tracking version of the task-based fMRI experiment to test whether eye movements differed systematically between conditions (these data could then be used to create an RDM based on eye movements and include this RDM in the fMRI analyses as a control model).*

We thank the reviewer for raising this important concern. Please see the first section of the *Response to the General Comments* at the beginning of this letter for a detailed discussion of this issue.

3) *One of the main results is the late and task-dependent representation of wall orientation in early visual cortex (V1). The late emergence of this response shows that it is not driven by the initial feedforward sweep but likely reflects an influence of spatial / feature-based attention or working memory (or eye movements, see above). If so, I find it hard to understand how V1 specifically "encodes" navigation-relevant properties (as the manuscript states) - that would be more a function of scene-selective regions. Rather, this could be more parsimoniously explained by a task-specific modulation of known V1 encoding properties: Attention to (or memory of) task-diagnostic locations in space and/or to orientations in the image could explain these findings. For example, to perform the task, participants would attend to wall orientation, and this is then reflected in V1 activity patterns, reflecting activity in orientation-selective neural populations.*

We thank the reviewer for raising this important point. Please see the second section of the *Response to the General Comments* at the beginning of this letter for a detailed discussion of this issue.

*Minor*

1) *Did some of the dimensions (e.g., wall distance) covary with other picture properties, such as depth-of-field?*

We thank the reviewer for raising this potential confound. Depth-of-field (DoF) is indeed a visual property that can correlate with perceived distance (Hafri et al., 2022). For the NSD dataset, which is based on the COCO image set, we acknowledge that DoF varies across images. However, it is not feasible to reliably disentangle optical DoF from other sources of image blur, such as motion blur, lens aberrations, or low resolution in unconstrained natural scenes. Importantly, our layout annotation method relies on the pinhole camera model that focuses on geometric structure and is theoretically independent of optical blur. In addition, our RSA analyses incorporated GIST and texture models that capture global spatial frequency distributions and local contrast variations, which are sensitive to blur-related image statistics. These control models therefore help mitigate potential confounds arising from DoF-related low-level visual differences.

For the Matterport3D experiments, DoF was effectively controlled at the data acquisition stage. All images were captured using a tripod-mounted camera rig with three color and three depth cameras pointing slightly up, horizontal, and slightly down (Chang et al., 2017). The cameras were configured with a far-focus setting (effectively at infinity), producing a large depth of field and images that remain sharp across the entire scene. Consequently, DoF is highly consistent across conditions in the Matterport3D experiments, and the observed neural representations of wall distance and orientation in these experiments cannot be attributed to DoF-related blur differences.

2) *The reported results are partial correlations; it would be relevant to know the correlations between the RDMs that are included in the analyses (e.g., GIST, texture, distance, orientation). This could be in the form of a correlation matrix, possibly in the Supp Material. For example, in Fig 7A, the negative correlation for orientation peaks at the same time (around 200 ms) as the positive correlation for distance, suggesting that these two models were related and that the negative correlation could be an artifact of the partial correlation method.*

We thank the reviewer for identifying this important issue. Please see the third section of the *Response to the General Comments* at the beginning of this letter for a detailed discussion of this issue.

**Reviewer 2**

1) *The relative-distance proxy is derived from area differences (not metric distance). The authors should quantify how this proxy relates to true distance on Matterport3D and show that results hold with alternative formulations of distance to demonstrate that the results are truly distance -related.*

We thank the reviewer for this important and constructive evaluation of our distance metric. We agree that it is essential to clarify how our area-based proxy relates to true metric distance and to demonstrate that our findings genuinely reflect distance-related representations rather than artifacts of image geometry.

The area difference provides a mathematically principled and robust estimate of relative distance within a fixed environmental context (see Appendix A of this letter for formal analysis), meaning that for any specific image where the room dimensions and camera settings are constant, the wall's size provides a consistent cue for its proximity. Under a horizontal camera pitch, the projected vertical extent of a wall in the image varies monotonically with its depth. This order-preserving property remains stable across moderate variations in pitch angle and is unlikely to be affected by the small roll angles present in the indoor scenes of the NSD dataset. Because this relationship may not hold under corner cases, we further evaluated the validity of the proxy empirically by comparing it against ground-truth metric distances in the Matterport3D dataset.

Across the five bins of one scene, we observed a strong correlation between the area difference and true metric distance: larger area differences consistently corresponded to shorter physical distances (Figure R5A). Furthermore, a correlation analysis pooling data across all bins of all images revealed a strong negative correlation between area differences and precise physical distances (r = -0.892) (Figure R5B). In the RSA framework, the RDM of the relative distance model exhibited a strong positive correlation with that of the precise distance model (r = 0.73). These empirical validations confirm that the proxy provides a reliable estimate of relative egocentric distance in realistic indoor environments.

However, within the NSD dataset, substantial variations in camera parameters and heights may weaken the correspondence between area difference and precise metric distance when comparisons are made across different images. Nevertheless, the monotonic relationship between these two metrics remains robust within any single image. We therefore term the area-based metric the “relative distance model”, as it addresses the question of which wall—the one in the left or right visual field—is closer to the observer.

In the revised manuscript, we implemented several changes to clarify concerns regarding the distance metric. First, we expanded the description of the relative distance model at its initial introduction in the Results section (p. 6). Second, we added a more detailed explanation of the precise distance model at its first appearance in the Results (p. 13), explicitly noting that “two images that exhibit similar side-wall area in 2D image plane may nonetheless correspond to substantially different physical distances in 3D space.” Third, we incorporated an additional paragraph in the Discussion (p. 20) to interpret the absence of significant precise-distance effects. In this section, we highlight that this finding “implies that the visual system may preferentially employ intuitive, heuristic-based spatial representations grounded in visual geometry, such as the relative image area occupied by surfaces.” We believe that clarifying the relationship between area-based proxies and precise metric distance is important, and that further work will be needed to fully characterize how these variables contribute to boundary representation.

**Figure R5**

Relationship between the relative distance model and precise distance model in the Matterport3D dataset. **A**, Within-image Pearson correlation. Each data point represents a single image. For each image, Pearson correlations were computed between the area-difference parameters across five bins in the relative distance model and the corresponding ground-truth distances in meters. **B**, Scatter plot of all bins of distance models across images. Each dot corresponds to a single bin from a single image, plotting the parameter from the relative distance model against the precise distance model.

*Since there is no result with metric distance, I'm worried that the effects in V1 can be due to other features that covary with the Distance measured as area difference. For instance, contrast in foveal area? Maybe smaller walls are more cluttered by objects or people? I think that the easier way would be to confirm the results with simple non-naturalistic images which include only walls without objects.*

We agree that the concern raised by the reviewer deserves further clarification. Consistent with standard practice in studies of scene-selective neural representations (e.g., Bonner & Epstein, 2017; Park et al., 2011), our primary analyses incorporated multiple control models—including GIST, texture, and semantic RDMs—to account for a broad range of non-navigational visual properties. The use of multiple control models reflects a deliberate balance between maintaining ecological validity with naturalistic stimuli and achieving rigorous quantitative control over image statistics. While no single model can exhaustively capture all visual features, jointly modeling these dimensions provides a principled baseline against which high-level layout information can be isolated from general image statistics.

Given the high degree of similarity between the relative and precise distance models, the non-significant findings for the precise distance model within the partial correlation analyses warrant a cautious interpretation. While both models aim to characterize wall distance, our results consistently showed a significant relationship between the relative distance model and neural activity in both full and partial correlation analyses. This contrast suggests that while wall distance is indeed encoded in the early visual cortex, the relative distance model serves as a more biologically plausible and effective descriptor of this representation. The lack of significant correlation for the precise distance model suggests that the visual system may prioritize more intuitive, heuristic-based representations of space derived from visual geometry rather than computing the objective, metric dimensions of the surrounding environment.

To directly address the reviewer’s specific hypotheses regarding potential confounds from contrast in foveal area and object clutter, we constructed two additional quantitative control models using the Matterport3D stimuli. For contrast in foveal area, we computed the standard deviation of pixel intensities within a central circular region (2° radius) for each image. Given that the mean maximum saccade distance in the Matterport3D MEG experiment was 1.330° (see the first section of the *Response to General Comments*), this measurement provides a conservative approximation of the contrast level in foveal area. For object clutter, we leveraged the instance labels provided in the Matterport3D dataset to quantify the number of objects within each of the five bins used in the relative distance model.

We then constructed RDMs for these two variables using Cityblock distance and compared them to all models included in the main analyses (Figure R6). The central contrast RDM showed the strongest similarity to the GIST model, as both models capture low-level image statistics. The object clutter RDM exhibited positive correlations with both the relative distance and precise distance models, which is expected because more distant walls typically encompass a larger spatial extent and thus contain more objects. Importantly, wall size itself is a geometric consequence of layout structure (distance and orientation) was not treated as a separate confounding variable. Instead, our focus was on quantifying how object clutter might covary with wall distance and orientation.

To evaluate whether these features could account for the observed neural effects, we incorporated the central contrast and object clutter RDMs as additional control variables in partial RSA analyses for both the Matterport3D fMRI and MEG experiments. As shown in Figures R7 and R8, although these control models shared some variance with other visual or layout features, the representations of boundary distance and orientation remained statistically significant after controlling for them. These results indicate that the primary findings cannot be explained solely by central contrast or object clutter.

Finally, the reviewer suggested the use of simplified, non-naturalistic stimuli (e.g., empty rooms without objects). While such stimuli offer maximal experimental control, they substantially reduce ecological validity and may not adequately capture the computations required to extract 3D structure from complex real-world environments. We therefore opted for a complementary approach: retaining naturalistic scenes while statistically controlling for potential confounds. This strategy provides a stringent and ecologically grounded test of whether neural representations reflect layout geometry beyond visual clutter and low-level image statistics. In the revised manuscript, we have added the results incorporating these two control models for the Matterport3D fMRI experiment to the Results (p. 13), Supplementary Methods, and Supplementary Figure 6.

**Figure R6**

Spearman correlations between the foveal contrast and object clutter models and all other models included in the main RSA analyses.

**Figure R7**

Partial RSA results for the Matterport3D fMRI experiment including fovea contrast and object clutter models. **A and C**, Results after incorporating the central contrast model during the layout discrimination task and texture discrimination task. **B and D**, Results after incorporating the object clutter model during the layout discrimination task and texture discrimination task. * *q* < 0.05; ** *q* < 0.01, *** *q* < 0.001

**Figure R8**

Partial RSA results for the Matterport3D MEG experiment including fovea contrast and object clutter models. **A and C**, Results after incorporating the foveal contrast model during the layout discrimination task and texture discrimination task. **B and D**, Results after incorporating the object clutter model during the layout discrimination task and texture discrimination task. Statistical testing procedures are consistent with those in Figure 7 of the main text.

2) *Another worry is in eye movements: Bigger nearby surfaces may attract fixations, modulating responses via attention rather than distance per se. The authors could quantify eye movements (e.g. number of fixations, average fixation length, etc.) using the DeepMReye software even if they did not collect eye movements by design.*

We thank the reviewer for raising this important concern. Please see the first section of the *Response to the General Comments* at the beginning of this letter for a detailed discussion of this issue.

3) *It is not clear why the number of visual ROIs considered diminishes in the second experiment. A strong justification is needed. Were the ROIs pre-registered? Results are the sames (in terms of statistics and multiple comparisons) if all the ROIs are considered?*

We thank the reviewer for the opportunity to clarify our ROI selection strategy and the apparent difference in the number of visual ROIs between experiments. Although the study was not formally pre-registered, ROI selection in both datasets was strictly hypothesis-driven and grounded in prior literature on navigational affordance and scene layout representation. Previous work has consistently focused on early visual cortex (EVC/V1) together with core scene-selective regions—including OPA, PPA, and RSC—as the principal nodes supporting the analysis of spatial layout and navigational structure (e.g., Bonner & Epstein, 2017; Henriksson et al., 2019; Lescroart & Gallant, 2019; Park & Park, 2020). Our ROI definitions were therefore determined a priori based on this established functional hierarchy rather than on statistical outcomes in the present dataset.

The difference in the number of ROIs between the NSD and Matterport3D fMRI experiment primarily reflects differences in available functional localizer data. The NSD dataset includes extensive retinotopic and functional mapping runs, allowing reliable delineation of multiple early visual areas (e.g., V1–hV4). In contrast, the Matterport3D fMRI experiment did not include full individual retinotopic mapping sessions. Consequently, we adopted a conservative anatomical-parcellation approach using FreeSurfer, which permits reliable identification of primary visual cortex (V1) but does not support precise subdivision of higher-order retinotopic areas (e.g., V2–V4) without functional mapping. To avoid introducing potentially unreliable ROI boundaries, we therefore restricted early visual analyses in the Matterport3D dataset to V1 alongside the core scene-selective ROIs (OPA, PPA, and RSC).

Importantly, this difference does not affect the interpretability of the results. The primary theoretical comparisons concern early visual cortex versus scene-selective regions, which are consistently represented across both datasets. Furthermore, the statistical procedures—including multiple-comparison correction—were applied consistently within each experiment according to the number of ROIs analyzed. In the revised manuscript, we have further clarified the ROI selection in the Methods section (p. 26 and 31).

**Reviewer 3**

1) *While the paper seem somewhat technically focused (e.g., quantification of 3D info), I found the paper to be well referenced and well situated within important theoretical frameworks in spatial cognition, such as vector-based coding of spatial layout (e.g., Bicanski & Burgess, 2020). The use of the Natural Scenes Dataset (NSD) is a great approach and sets the paper apart from other more controlled or game-based scene approaches. Because NSD does not include ground-truth 3D layout annotations, the authors' development of a computer vision pipeline to reconstruct 3D scene structure is crucial and needed. The validation on the Matterport3D dataset, which includes known camera parameters, provides confidence in the approach. One question that could be addressed more clearly is whether the method generalizes to outdoor environments, or whether its assumptions are specific to indoor scenes. Clarifying this would help contextualize the scope and limitations of the approach. Related to above, I also wonder if the authors make the 3D analysis codes available.*

We thank the reviewer for this constructive question regarding the generalizability and practical scope of our reconstruction approach. Our camera calibration method relies on identifying at least two sets of parallel line segments that are orthogonal to each other and parallel to the ground. In indoor environments, these constraints are typically satisfied by structural elements such as wall intersections, baseboard, or rectilinear furniture. Importantly, the method is not intrinsically limited to indoor scenes. It can generalize to urban outdoor environments where man-made structures provide stable rectilinear geometry—for example, street-view scenes containing building facades, sidewalks, or road markings that yield reliable parallel edges and vanishing points.

However, the layout reconstruction approach is based on the Manhattan World assumption, which models environments as composed of planar surfaces that are mutually orthogonal. As a consequence, its applicability is reduced in natural outdoor settings that lack rectilinear structure (e.g., forests, mountainous terrain, or open fields), or in environments characterized by curved layouts or irregular geometries. Extending the current framework to such scenes would likely require additional constraints. We have added a paragraph in the revised Discussion (p. 18) to explicitly clarify applicability of the method in outdoor environments.

Regarding reproducibility, all analysis code used for 3D layout reconstruction and camera calibration has been made publicly available on OSF at <https://osf.io/uxwr4/>.

2) *The authors' representational similarity analysis (RSA) reveals an interesting dissociation between the neural encoding of distance and orientation. After statistically controlling for low-, mid-, and high-level 2D image statistics (GIST, Portilla-Simoncelli, object2vec), early visual cortex continues to track relative distance, whereas hV4, OPA, and PPA selectively track boundary orientation. One thing that I was not clear of was how the authors controlled for the low-level information to set it apart from layout information. Did the authors use regression modeling, or some other kind of orthogonalization?*

To isolate layout-related information from low-level visual features, we employed partial Spearman correlations within an RSA framework rather than regression-based modeling of feature spaces. Partial RSA is a well-established and robust approach for disentangling overlapping representational structures in complex naturalistic stimuli. For additional methodological details and representative studies adopting this approach, please refer to the third section of the *Response to the General Comments* at the beginning of this letter.

3) *The finding that semantic features showed partial correlations even in early visual areas is interesting and deserves a sentence or two of interpretation, as it seems to underscore the extent to which even early visual cortex is influenced by high-level structure in naturalistic inputs.*

We thank the reviewer for the suggestion. We agree that the presence of significant partial correlations for semantic features in early visual cortex is noteworthy, and we have added brief interpretational remarks in the Results section (p. 7) of the revised manuscript.

From a methodological perspective, it is inherently difficult to fully orthogonalize low-level visual features and high-level semantic categories in naturalistic stimuli. Although the GIST and texture models capture many global and local image statistics, they may not fully account for all local spatial contours or mid-level visual features (e.g., diagnostic shape fragments) that systematically covary with object categories. As a result, some variance that is visual in nature may "leak" into the semantic model if that model better captures the consistent morphological patterns of certain indoor objects. Under this interpretation, partial correlation with semantic model in early visual cortex may reflect complex visual statistics beyond the descriptive power of standard low-level models.

At the same time, emerging evidence suggests that early visual cortex can be modulated by semantic information during natural vision. For example, analyses using the NSD dataset have demonstrated that semantic dimensions can explain variance even in early visual cortex (Doerig et al., 2025). Similarly, research using the THINGS dataset has shown that behavior-derived object dimensions predict neural responses across both category-selective cortex and early visual cortex (Contier et al., 2024). These findings raise the possibility that top-down influences or recurrent processing may contribute to semantic sensitivity in early visual cortex under naturalistic viewing conditions.

Nevertheless, we remain cautious in attributing these effects to explicit semantic representations in early visual cortex. Our NSD subset consisted exclusively of indoor scenes and therefore sampled only a restricted portion of the broader semantic space. Importantly, the three control models behaved as expected across the cortical hierarchy: the GIST model showed the strongest effects in early visual areas, whereas the semantic model exhibited stronger correlations in category-selective regions than in V1–V4. Together, these validation patterns provide confidence that the control models accounted for the majority of irrelevant visual and semantic variance, allowing us to isolate the unique contribution of the 3D layout representations.

4) *The dissociation between 3D layout features (e.g., distance, orientation) and self-pose features (pitch, roll) is interesting but seems under-described in the paper. While distance and orientation is intuitively clear, self-pose features are less intuitive and may benefit from additional clarification in the introduction. Related to this point, I wonder whether the authors view self-pose features as distinct viewer-centered/egocentric parameters while view the 3D layout features as allocentric parameters.*

We thank the reviewer for raising the question regarding the conceptual framing of self-pose and 3D layout representations. Self-pose parameters (pitch and roll) describe the observer’s state—specifically the facing direction of the observer (or camera) relative to the environment’s horizontal and vertical axes. They address the question: How is my head/body tilted relative to the scene? In contrast, the 3D layout features characterize the spatial geometry of environment relative to the observer, addressing where are the boundaries in the surrounding environment. Importantly, in the present study both classes of features are inherently egocentric, as they are directly related to the visual input of a first-person perspective. Therefore, we do not conceptualize layout features as allocentric representations per se; rather, they reflect egocentric descriptions of environmental geometry.

Despite sharing an egocentric reference frame, the two feature classes serve distinct functional roles in navigation. Self-pose is primarily related to stabilizing perception and maintaining alignment between vestibular and visual signals, whereas layout features such as boundary distance and orientation provide the geometric scaffolding necessary for path planning. Consistent with this conceptual distinction, our computational models are mathematically dissociated: the boundary orientation model is defined from a top-down geometric projection that remains invariant to changes in pitch and roll, and the relative distance model is largely robust to self-pose variations (see Appendix A of this letter for formal analysis).

Finally, the distinct neural signatures observed for self-pose and layout features across experiments further support their functional separation despite a shared egocentric basis. Following the reviewer’s suggestion, we have provided additional clarification of self-pose features in the Results (p. 9) and expanded the Discussion (p. 19-20) to more explicitly address the dissociation between these two egocentric representations.

5) *The switch to decoding methods for self-pose features (rather than RSA, used elsewhere in the paper) would benefit from a justification.*

We thank the reviewer for raising this important methodological question. The use of decoding analyses for self-pose features was motivated by the representational structure of these variables. In contrast to the multi-dimensional geometric structures of 3D layout, self-pose parameters (pitch and roll) are continuous, single-dimensional signals. Decoding approaches are particularly appropriate for such parametric variables because they allow direct quantification of how accurately stimulus information can be recovered from neural activity patterns. By comparison, RSA is more suited to testing structured hypotheses about representational geometry across complex feature spaces, which was the primary goal of our layout analyses.

Importantly, the choice of decoding for self-pose was not intended to introduce a different analytical standard but rather to align the analysis method with the structure of the modeled feature space. To directly address the reviewer’s concern regarding methodological consistency and potential interactions between self-pose and layout representations, we conducted additional control analyses in which self-pose RDMs were included as covariates within the layout RSA framework.

As shown in Figure R9, the significant representations of boundary distance and orientation remained unchanged after controlling for self-pose. Critically, RSA analyses applied to the pitch model produced patterns in scene-selective regions that were consistent with the original decoding results.

**Figure R9**

Control RSA including self-pose parameters in the NSD experiment. Partial correlation analyses were conducted including self-pose, visual, and 3D layout models. * *q* < 0.05; ** *q* < 0.01, *** *q* < 0.001

6) *In my opinion, one of the most interesting parts of the paper was the demonstration that encoding of distance and orientation is modulated by task demands. The comparison between a texture discrimination task and a layout discrimination task shows that OPA exhibit enhanced correlations with layout-relevant features only when spatial judgments are required. This challenges the prevailing assumption that distance and orientation are exclusively bottom-up properties inherent to the image. Instead, the results strongly suggest top-down modulation in spatial layout processing. The authors' discussion points in this direction, but I believe the manuscript would be strengthened by developing this point further. In particular, it would be useful to elaborate on why some regions show task-dependent enhancement while others remain task-invariant, and also elaborate on mechanism of how the task-specific effects might influence these regions. Theoretically strengthening this results would help increase the significance of the findings.*

We thank the reviewer for highlighting the theoretical importance of the task-modulation effects. We agree that this aspect substantially strengthens the implications of our findings, and we have expanded the Discussion (p. 19) in the revised manuscript to provide a more explicit mechanistic account of task-dependent spatial layout processing along the visual hierarchy.

First, our results suggest a hierarchical framework in which distinct layout features are processed at different stages of the visual hierarchy. Relative distance information emerges rapidly in early visual cortex and remains largely task-invariant, consistent with automatic extraction of spatial structure from visual input. In contrast, boundary orientation arises later and is primarily represented in higher-level scene-selective regions. We propose that orientation constitutes a more abstract and computationally efficient descriptor of scene geometry, as it provides a stable structural scaffold that is relatively invariant to the observer’s position. Computationally, extracting orientation from depth structure may involve operations analogous to estimating spatial derivatives of the distance field, which require integrative processing across multiple spatial locations. This higher-order computation may account for both the delayed temporal profile and the stronger sensitivity to task demands.

Second, the differential task modulation reflects functional specialization within the visual hierarchy. We observed late-stage task-dependent enhancement in early visual cortex (>500 ms), which likely reflects feedback-driven refinement. Such late effects are consistent with recurrent processing models in which higher-level areas (e.g., OPA and parietal regions) provide top-down signals that enhance task-relevant spatial features in earlier visual areas. Please refer to the second section of the *Response to the General Comments* at the beginning of this letter for a detailed discussion about this issue.

**Appendix A**

We analyze a column-wise statistic defined as the difference between the number of wall pixels and the number of horizontal-surface pixels (ceiling and/or floor) within each image column. The aim is to clarify the geometric conditions under which this statistic reflects wall depth.

For a given image column x, we define the column-wise length-difference statistic as

$$S\left( x \right)=wall-(ceiling+floor)$$

**A.1 Zero roll and zero pitch: metric depth**

Let $y_{c}(x)$ and $y_{f}(x)$ denote the ceiling–wall and wall–floor boundaries in column *x*, respectively. Given an image height *H* in the pixel coordinate, the statistic can be expressed as

$$S\left( x \right)=2\left( y_{c}\left( x \right) - y_{f}\left( x \right) \right)-H$$

Assuming the ceiling and floor are separated by a vertical height difference of $Y_{0}$, perspective projection yields

$$y_{c}\left( x \right) - y_{f}\left( x \right)=f\frac{Y_{0}}{Z_{w}(x)}$$

where $Z_{w}(x)$ denotes the depth of the wall intersected by column *x* and *f* is the focal length in pixels. Thus, under these conditions, the statistic provides metric (inverse-depth) information.

**A.2 Nonzero pitch and roll: preservation of ordinal depth**

We next allow the camera pitch angle $\phi$ and roll angle $\theta$ to vary. Under conditions of non-zero pitch and roll, the proposed statistic no longer corresponds to a single depth value within a column. Nevertheless, it varies monotonically with the depths of the wall–ceiling and/or wall–floor intersections in that column.

For a horizontal plane at world height *Y*, its image projection *y* as a function of depth *Z* is given by

$$y\left( Z \right) =f\frac{Xsin\theta+Ycos\phi cos\theta-Zcos\theta sin\phi}{Ysin\phi+Zcos\phi}$$

Differentiating with respect to *Z*,

$$\frac{dy}{dZ}=-f\frac{Ycos\theta+Xcos\phi sin\theta}{{(Ysin\phi+Zcos\phi)}^{2}}$$

In our NSD indoor dataset, the roll angle are typically within 10°. Therefore, the effect of roll can be considered negligible.

$$\frac{dy}{dZ}\approx-\frac{fY}{{(Ysin\phi+Zcos\phi)}^{2}}$$

As long as the plane remains visible, the denominator is strictly positive and *Y* is constant, implying that $y\left( Z \right)$ is a strictly monotonic function with respect to depth. Because ceiling heights are positive and the floor heights are negative in image coordinates, the statistic $S\left( x \right)$ decreases monotonically as the wall depth increases.

**A.3 Columns without horizontal surfaces**

In some columns, neither ceiling nor floor may be visible, and the column consists entirely of wall pixels. In this case, $S\left( x \right)$ degenerates to a constant value and cannot distinguish depth differences between such columns.

However, they correspond to walls located closer to the camera than columns in which at least one horizontal plane remains visible.

**A.4 Mixed visibility across columns: limits of global ordering**

A more subtle situation arises when different columns within the same image contain different sets of visible planes. For example, there may be columns *A* where ceiling, wall, and floor are all visible and columns *B* where only wall and floor are visible. In this case, the statistic takes different functional forms:

$$S_{A}\left( x \right)=2\left( y_{c}\left( x \right) - y_{f}\left( x \right) \right)-H$$

$$S_{B}\left( x \right)=2y_{f}\left( x \right)-H$$

Although both $S_{A}\left( x \right)$ and $S_{B}\left( x \right)$ are individually monotonic functions with respect to wall depth, they do not share a common monotonic mapping. As a result, numerical comparison across column types does not guarantee a consistent depth ordering.

**A.5 Summary**

In summary, the proposed column-wise statistic provides a valid proxy of wall distance in following conditions. First, the statistic reflects metric depth under zero pitch and consistent visibility of horizontal planes. Second, the statistic reflects ordinal depth under nonzero pitch, provided at least one horizontal plane is visible. However, the statistic reflects only coarse near–far distinctions for columns without horizontal planes and no guaranteed global ordering when horizontal-plane visibility differs across columns.

**References**

Allen, E. J., St-Yves, G., Wu, Y., Breedlove, J. L., Prince, J. S., Dowdle, L. T., Nau, M., Caron, B., Pestilli, F., Charest, I., Hutchinson, J. B., Naselaris, T., & Kay, K. (2022). A massive 7T fMRI dataset to bridge cognitive neuroscience and artificial intelligence. *Nature Neuroscience*, *25*(1), 116–126. https://doi.org/10.1038/s41593-021-00962-x

Bainbridge, W. A., Hall, E. H., & Baker, C. I. (2021). Distinct Representational Structure and Localization for Visual Encoding and Recall during Visual Imagery. *Cerebral Cortex*, *31*(4), 1898–1913. https://doi.org/10.1093/cercor/bhaa329

Bonner, M. F., & Epstein, R. A. (2017). Coding of navigational affordances in the human visual system. *Proceedings of the National Academy of Sciences*, *114*(18), 4793–4798. https://doi.org/10.1073/pnas.1618228114

Bonner, M. F., & Epstein, R. A. (2018). Computational mechanisms underlying cortical responses to the affordance properties of visual scenes. *PLOS Computational Biology*, *14*(4), e1006111. https://doi.org/10.1371/journal.pcbi.1006111

Chang, A., Dai, A., Funkhouser, T., Halber, M., Nießner, M., Savva, M., Song, S., Zeng, A., & Zhang, Y. (2017). *Matterport3D: Learning from RGB-D Data in Indoor Environments* (arXiv:1709.06158). arXiv. https://doi.org/10.48550/arXiv.1709.06158

Cichy, R. M., Kriegeskorte, N., Jozwik, K. M., van den Bosch, J. J. F., & Charest, I. (2019). The spatiotemporal neural dynamics underlying perceived similarity for real-world objects. *NeuroImage*, *194*, 12–24. https://doi.org/10.1016/j.neuroimage.2019.03.031

Contier, O., Baker, C. I., & Hebart, M. N. (2024). Distributed representations of behaviour-derived object dimensions in the human visual system. *Nature Human Behaviour*, 1–15. https://doi.org/10.1038/s41562-024-01980-y

Dijkstra, N., Bosch, S. E., & van Gerven, M. A. J. (2019). Shared Neural Mechanisms of Visual Perception and Imagery. *Trends in Cognitive Sciences*, *23*(5), 423–434. https://doi.org/10.1016/j.tics.2019.02.004

Dijkstra, N., Mostert, P., Lange, F. P. de, Bosch, S., & van Gerven, M. A. (2018). Differential temporal dynamics during visual imagery and perception. *eLife*, *7*, e33904. https://doi.org/10.7554/eLife.33904

Doerig, A., Kietzmann, T. C., Allen, E., Wu, Y., Naselaris, T., Kay, K., & Charest, I. (2025). High-level visual representations in the human brain are aligned with large language models. *Nature Machine Intelligence*, *7*(8), 1220–1234. https://doi.org/10.1038/s42256-025-01072-0

Dövencioğlu, D., Ban, H., Schofield, A. J., & Welchman, A. E. (2013). Perceptual Integration for Qualitatively Different 3-D Cues in the Human Brain. *Journal of Cognitive Neuroscience*, *25*(9), 1527–1541. https://doi.org/10.1162/jocn_a_00417

Engbert, R., & Kliegl, R. (2003). Microsaccades uncover the orientation of covert attention. *Vision Research*, *43*(9), 1035–1045. https://doi.org/10.1016/S0042-6989(03)00084-1

Epstein, R. A., & Baker, C. I. (2019). Scene Perception in the Human Brain. *Annual Review of Vision Science*, *5*(1), 373–397. https://doi.org/10.1146/annurev-vision-091718-014809

Fang, F., Boyaci, H., Kersten, D., & Murray, S. O. (2008). Attention-Dependent Representation of a Size Illusion in Human V1. *Current Biology*, *18*(21), 1707–1712. https://doi.org/10.1016/j.cub.2008.09.025

Georgieva, S. S., Todd, J. T., Peeters, R., & Orban, G. A. (2008). The Extraction of 3D Shape from Texture and Shading in the Human Brain. *Cerebral Cortex*, *18*(10), 2416–2438. https://doi.org/10.1093/cercor/bhn002

Gerardin, P., Kourtzi, Z., & Mamassian, P. (2010). Prior knowledge of illumination for 3D perception in the human brain. *Proceedings of the National Academy of Sciences*, *107*(37), 16309–16314. https://doi.org/10.1073/pnas.1006285107

Giari, G., Leonardelli, E., Tao, Y., Machado, M., & Fairhall, S. L. (2020). Spatiotemporal properties of the neural representation of conceptual content for words and pictures – an MEG study. *NeuroImage*, *219*, 116913. https://doi.org/10.1016/j.neuroimage.2020.116913

Hafed, Z. M., & Clark, J. J. (2002). Microsaccades as an overt measure of covert attention shifts. *Vision Research*, *42*(22), 2533–2545. https://doi.org/10.1016/S0042-6989(02)00263-8

Hafri, A., Wadhwa, S., & Bonner, M. F. (2022). Perceived Distance Alters Memory for Scene Boundaries. *Psychological Science*, 09567976221093575. https://doi.org/10.1177/09567976221093575

Harrison, S. A., & Tong, F. (2009). Decoding reveals the contents of visual working memory in early visual areas. *Nature*, *458*(7238), 632–635.

He, D., Mo, C., Wang, Y., & Fang, F. (2015). Position shifts of fMRI-based population receptive fields in human visual cortex induced by Ponzo illusion. *Experimental Brain Research*, *233*(12), 3535–3541. https://doi.org/10.1007/s00221-015-4425-3

Henriksson, L., Mur, M., & Kriegeskorte, N. (2019). Rapid Invariant Encoding of Scene Layout in Human OPA. *Neuron*, *103*(1), 161-171.e3. https://doi.org/10.1016/j.neuron.2019.04.014

Jia, K., Li, Y., Gong, M., Huang, H., Wang, Y., & Li, S. (2021). Perceptual learning beyond perception: Mnemonic representation in early visual cortex and intraparietal sulcus. *Journal of Neuroscience*, *41*(20), 4476–4486.

Kaiser, D., Jacobs, A. M., & Cichy, R. M. (2022). Modelling brain representations of abstract concepts. *PLOS Computational Biology*, *18*(2), e1009837. https://doi.org/10.1371/journal.pcbi.1009837

Kwak, Y., & Curtis, C. E. (2022). Unveiling the abstract format of mnemonic representations. *Neuron*, *110*(11), 1822-1828.e5. https://doi.org/10.1016/j.neuron.2022.03.016

Lescroart, M. D., & Gallant, J. L. (2019). Human Scene-Selective Areas Represent 3D Configurations of Surfaces. *Neuron*, *101*(1), 178-192.e7. https://doi.org/10.1016/j.neuron.2018.11.004

Lu, Z., & Golomb, J. (2025). Human EEG and artificial neural networks reveal disentangled representations and processing timelines of object real-world size and depth in natural images. *eLife*, *13*, RP98117. https://doi.org/10.7554/eLife.98117

Murray, S. O., Boyaci, H., & Kersten, D. (2006). The representation of perceived angular size in human primary visual cortex. *Nature Neuroscience*, *9*(3), 429–434. https://doi.org/10.1038/nn1641

Park, J., & Park, S. (2020). Coding of Navigational Distance and Functional Constraint of Boundaries in the Human Scene-Selective Cortex. *The Journal of Neuroscience*, *40*(18), 3621–3630. https://doi.org/10.1523/JNEUROSCI.1991-19.2020

Park, S., Brady, T. F., Greene, M. R., & Oliva, A. (2011). Disentangling Scene Content from Spatial Boundary: Complementary Roles for the Parahippocampal Place Area and Lateral Occipital Complex in Representing Real-World Scenes. *Journal of Neuroscience*, *31*(4), 1333–1340. https://doi.org/10.1523/JNEUROSCI.3885-10.2011

Pasternak, T., & Greenlee, M. W. (2005). Working memory in primate sensory systems. *Nature Reviews Neuroscience*, *6*(2), 97–107.

Rademaker, R. L., Chunharas, C., & Serences, J. T. (2019). Coexisting representations of sensory and mnemonic information in human visual cortex. *Nature Neuroscience*, *22*(8), Article 8. https://doi.org/10.1038/s41593-019-0428-x

Reddy, L., Cichy, R. M., & VanRullen, R. (2021). Representational Content of Oscillatory Brain Activity during Object Recognition: Contrasting Cortical and Deep Neural Network Hierarchies. *eNeuro*, *8*(3). https://doi.org/10.1523/ENEURO.0362-20.2021

Serences, J. T., Ester, E. F., Vogel, E. K., & Awh, E. (2009). Stimulus-specific delay activity in human primary visual cortex. *Psychological Science*, *20*(2), 207–214.

Welchman, A. E. (2016). The Human Brain in Depth: How We See in 3D. *Annual Review of Vision Science*, *2*(1), 345–376. https://doi.org/10.1146/annurev-vision-111815-114605

Welchman, A. E., Deubelius, A., Conrad, V., Bülthoff, H. H., & Kourtzi, Z. (2005). 3D shape perception from combined depth cues in human visual cortex. *Nature Neuroscience*, *8*(6), 820–827. https://doi.org/10.1038/nn1461

Xie, S., Kaiser, D., & Cichy, R. M. (2020). Visual Imagery and Perception Share Neural Representations in the Alpha Frequency Band. *Current Biology*, *30*(13), 2621-2627.e5. https://doi.org/10.1016/j.cub.2020.04.074

Xu, W., Li, X., Parviainen, T., & Nokia, M. (2024). Neural correlates of retrospective memory confidence during face–name associative learning. *Cerebral Cortex*, *34*(5). https://doi.org/10.1093/cercor/bhae194

Yuval-Greenberg, S., Merriam, E. P., & Heeger, D. J. (2014). Spontaneous Microsaccades Reflect Shifts in Covert Attention. *Journal of Neuroscience*, *34*(41), 13693–13700. https://doi.org/10.1523/JNEUROSCI.0582-14.2014
